# Three People With Recurrent Nephrolithiasis and Heterozygous *ABCC6* Mutations

**DOI:** 10.1016/j.xkme.2026.101271

**Published:** 2026-01-22

**Authors:** Douglas Farrell, Jaime Uribarri, Tessa R. Pitman, Mark Lebwohl, Joshua L. Rein

**Affiliations:** 1 Barbara T. Murphy Division of Nephrology, Department of Medicine, Icahn School of Medicine at Mount Sinai, New York, NY; 2 Transplant and Renal Genetics, Natera Inc, San Carlos, CA; 3 Department of Dermatology, Icahn School of Medicine at Mount Sinai, New York, NY; 4 Renal Section, Department of Medicine, James J. Peters Veterans Affairs Medical Center, Bronx, NY

**Keywords:** Nephrolithiasis, kidney stone, calcium oxalate, calcification, pseudoxanthoma elasticum, genetic testing

## Abstract

Monogenic causes of nephrolithiasis and nephrocalcinosis are relatively common but underdiagnosed. Pseudoxanthoma elasticum (PXE) is an autosomal recessive disease that causes progressive ectopic calcium phosphate deposits throughout the body. PXE results from homozygous mutations in the ATP-binding cassette subfamily C member 6 (*ABCC6*) gene, which encodes an ATP transporter that is predominantly expressed in the liver but also expressed in the kidney proximal tubule. ABCC6 transports ATP extracellularly, where ectonucleotide pyrophosphatase/phosphodiesterase 1 metabolizes ATP into AMP and pyrophosphate (PP_i_), an inhibitor of calcium crystallization. Loss-of-function mutations in *ABCC6* are associated with low serum PP_i_ levels, leading to ectopic calcifications. PXE is associated with an increased risk of nephrolithiasis, but it is currently unknown if heterozygotes are also at risk. Herein, we presented 3 patients with recurrent nephrolithiasis who had relatively unremarkable risk factors but were found to have heterozygous mutations in *ABCC6—*patient 1 c.1685T>C (p.Met562Thr); patient 2 c.933C>A (p.Phe311Leu); and patient 3 c.3413G>A (p.Arg1138Gln). We proposed that heterozygous *ABCC6* mutations are an unrecognized risk factor for nephrolithiasis. Development of a clinical assay to measure urinary PP_i_ may help identify people at risk of nephrolithiasis, elucidate the underlying mechanisms of recurrent nephrolithiasis, and potentially identify a therapeutic target to reduce stone burden.

## Introduction

Pseudoxanthoma elasticum (PXE) is an autosomal recessive disease that causes progressive ectopic calcium phosphate deposits throughout the body, leading to skin lesions, angioid streaks, and arterial calcification.^1^ PXE results from homozygous mutations in the ATP-binding cassette sub-family C member 6 (*ABCC6*) gene, which encodes an ATP transporter that is predominantly expressed in the liver but also expressed in the kidney proximal tubule.^2^^,^^3^ ABCC6 transports ATP extracellularly, where ectonucleotide pyrophosphatase/phosphodiesterase 1 (ENPP-1) metabolizes ATP into AMP and pyrophosphate (PP_i_), an inhibitor of calcium crystallization. Loss-of-function mutations in *ABCC6* are associated with low serum PP_i_ levels, leading to the ectopic calcification characteristic of PXE.^4^ Heterozygous mutations can also cause ectopic calcifications of the vasculature and abdominal organs, including the kidney.^5^, ^6^, ^7^ PXE is associated with an increased risk of nephrolithiasis, but it is currently unknown if heterozygotes are also at risk.^4^ Herein, we presented 3 patients with recurrent nephrolithiasis from 1 general nephrology outpatient clinic (J.L.R.), who had relatively unremarkable risk factors, except for family histories of nephrolithiasis, but were found to have heterozygous mutations in *ABCC6* during routine clinical care. These 3 patients were the only ones to be referred for genetic testing related to recurrent nephrolithiasis. We proposed that heterozygous *ABCC6* mutations are an unrecognized risk factor for nephrolithiasis.

## Case Report

### Case 1

**Figure 1:**
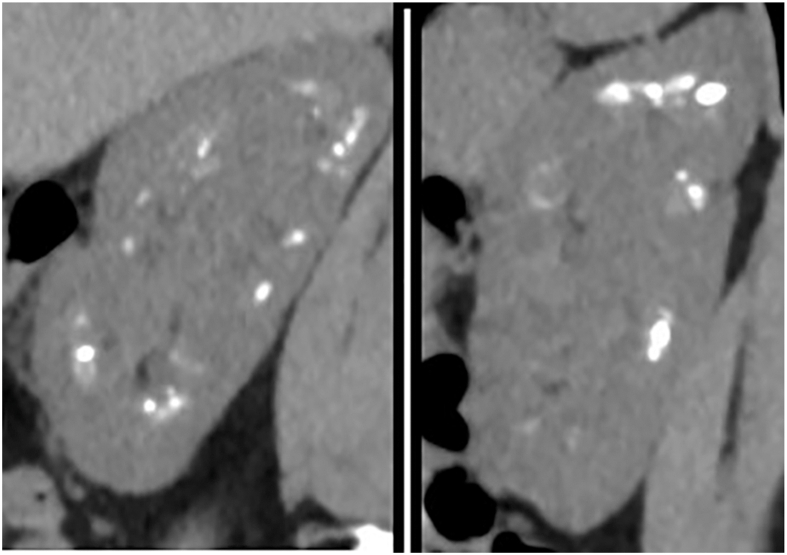
Non-contrast sagittal abdominal computed tomography images for Patient 1 revealed nephrocalcinosis.

A 45-year-old woman was referred for recurrent nephrolithiasis and nephrocalcinosis (Fig 1). Family history was significant for her mother, who also had recurrent nephrolithiasis. Laboratory workup was significant for an elevated 1,25-dihydroxyvitamin D level (73.8-116.0 [mean level 95.9] [normal reference range, 24.8-81.5] pg/mL), borderline hypercalciuria (urine calcium level 161-228 [mean level 202] [normal reference range, <250 for males and <200 for females] mg/24 h), hypocitraturia (urine citrate level 276-469 [mean level 397] [normal reference range, >450 for males and >550 for females] mg/24 h), normal serum bicarbonate level (22-26 meq/L), and a urine pH consistently > 6.0 (Tables 1 and 2). She had intermittent adherence to potassium bicarbonate 25 mEq twice daily, which was started after the first 24-hour urine. Stone analysis indicated 100% calcium phosphate. Because her presentation was consistent with an incomplete distal renal tubular acidosis, genetic testing was performed using the Natera Renasight kidney gene panel, which revealed a heterozygous variant of uncertain significance (VUS) in *ABCC6* (c.1685T>C (p.Met562Thr) rs72653775) with a Rare Exome Variant Ensemble Learner (REVEL) score of 0.84.^8^ An axillary skin biopsy and retinal examination respectively did not show any cutaneous calcification or angioid streaks.

**Table 1:** Relevant Ranges for Vital Signs and Spot Laboratory Tests Over Multiple Clinical Encounters for the 3 Described Cases

Vital signs and laboratory tests	Case 1	Case 2	Case 3
Weight, kg	49-55	60-65	59-62
Systolic blood pressure, mmHg	97-129	134-151	112-141
Diastolic blood pressure, mmHg	65-91	85-104	74-93
Serum HCO_3_^−^, mmol/L	21.6-26.4	26.3-29.3	22-26.9
Serum Ca^2+^, mg/dL	8.5-9.8	8.9-9.7	9.2-9.8
Serum P, mg/dL	3.0-3.3	2.6-3.2	3.3-4.0
iPTH, pg/mL	28-33	53	14
25-OH Vitamin D, ng/mL	28-32	30	48.7, 79.2
1,25-OH Vitamin D, pg/mL	73.8-116.0	57.9	70.9, 99.6
Serum Cr, mg/dL	0.6-0.7	1.1-1.2	0.7-0.8
Urine pH, -log[H^+^]	5.0-8.0	6.0-8.5	6.0-7.0

Ca^2+^, calcium; Cr, creatinine; HCO3^−^, bicarbonate; iPTH, intact parathyroid hormone; P, phosphorus.

**Table 2:** The 24-hour Urine Collections for 3 Described Patients During Evaluation for Recurrent Nephrolithiasis

24-h urine	Reference range	Case 1					Case 2						Case 3
Date		Sample 1	Sample 2	Sample 3	Sample 4	Sample 5	Sample 1	Sample 2	Sample 3	Sample 4	Sample 5	Sample 6	Sample 1
Total volume, (mL)	500-4,000	2,400	1,570	2,250	2,480	3,240	1,090	1,180	1,570	2,530	1,050	1,670	2,350
pH,−log[H^+^]	5.8-6.2	6.7	6.1	6.7	6.5	6.7	6.2	5.7	6.7	6.5	5.8	6.7	6.5
Creatinine, mg	500-2,000	1,087	775	934	987	987	1,949	1,814	1,228	1,344	1,335	1,344	851
Ca^2+^, mg	male < 250 female < 200	228	228	218	161	305	221	203	150	128	113	89	68
P_i_	600-1,200	295	347	534	379	439	886	1,057	691	822	666	559	320
Uric acid, mg	male < 800 female < 750	310	254	366	328	329	556	667	496	612	504	643	364
Na^+^, mEq	50-150	79	77	99	63	136	104	119	103	121	75	66	92
K^+^, mEq	20-100	30	32	51	46	51	48	39	44	55	33	54	51
Cl^–^, mEq	70-250	67	96	109	74	146	94	122	92	110	69	66	94
NH_4_^+^, mEq	15-60	17	20	15	17	19	37	42	24	29	29	29	15
Oxalate, mg	20-40	19	14	23	23	17	28	24	24	31	21	16	9
Citrate, mg	male > 450 female > 550	295	276	445	469	428	502	304	367	332	293	336	449
Osmolality, mOsm/kg	300-900	177	-	-	-	-	-	-	-	-	-	-	195
SO_4_^2–^, mEq	20-80	17	15	15	12	27	44	36	23	32	28	27	14
Mg^2+^, mg	30-120	82	69	82	48	64	107	107	61	65	52	48	33

Ca^+^, calcium; Cl^−^, chloride; P_i_, inorganic phosphate; K^+^, potassium; Mg^2+^, magnesium; Na^+^, sodium; NH_4_^+^, ammonium; SO_4_^2−^, sulfate.

### Case 2

A 35-year-old man was referred for recurrent nephrolithiasis. His history was significant for more than 20 lifetime occurrences of symptomatic nephrolithiasis. Family history for nephrolithiasis was significant for his father, paternal uncle, and a paternal first-degree cousin, while his mother, sister, paternal half-sister, paternal grandmother, 3 paternal aunts, 1 paternal uncle, and the first-degree cousin’s sister did not have stones.

Laboratory workup was only significant for relatively low 24-hour urine volume (1,090-2,530 [mean 1,515] mL) on 1 occasion and hypocitraturia (urine citrate level 293-502 [mean 356] [normal reference range, >450 for males and >550 for females] mg/24 h) (Tables 1 and 2). He was advised to modify his diet, though he was unable to adequately increase his water intake. An abdominal computed tomography scan acquired 2 months before the initial consultation showed 3 right-sided stones up to 5 mm with associated moderate hydroureteronephrosis and an additional 3 mm bladder stone. Genetic analysis using Natera Renasight identified an *ABCC6* heterozygous VUS: c.933C>A (p.Phe311Leu) with a REVEL score of 0.755. He was also heterozygous for a pathogenic mutation in *PLG*, in which biallelic deleterious mutations cause familial plasminogen deficiency that can be associated with nephrolithiasis. However, our patient did not have chronic mucosal pseudomembranous lesions or inflammation caused by abnormal fibrin degradation as would be clinically expected.

### Case 3

A 24-year-old woman was referred for recurrent nephrolithiasis. Her history was significant for hypertension controlled on a calcium channel blocker and angiotensin-2 receptor blocker. She had yearly recurrences of symptomatic nephrolithiasis since she was 2 years of age. Family history was significant for nephrolithiasis in her paternal grandmother and paternal great-aunt, whereas her father, paternal half-brother, and entire maternal side did not have it. Laboratory workup was significant for an elevated 1,25-dihydroxyvitamin D level (70.9-99.6 [mean level 85.25] [normal reference range, 24.8-81.5] pg/mL) (Tables 1 and 2). Dedicated stone imaging was unavailable, but abdominal magnetic resonance imaging did not reveal any kidney abnormalities. Stone analysis showed a composition of 90% calcium oxalate and 10% calcium phosphate. Although no immediate family members had a history of nephrolithiasis, her age at disease onset and longitudinal stone burden prompted genetic testing. Genetic analysis with Natera Renasight revealed heterozygosity for a pathogenic variant in *ABCC6* (c.3413G>A (p.Arg1138Gln) rs60791294) with a REVEL score of 0.958, in addition to a heterozygous VUS in *SLC34A3* (REVEL = 0.089). Similarly with *ABCC6*, heterozygous *SLC34A3* variants currently have limited evidence relating to nephrolithiasis and nephrocalcinosis risk, and this mutation is considered clinically insignificant.^9^, ^10^, ^11^ She also did not have hypophosphatemia nor hypercalciuria that are seen with hypophosphatemic rickets.

## Discussion

Monogenic causes of nephrolithiasis and nephrocalcinosis are relatively common but underdiagnosed.^12^ Risk factors for nephrolithiasis, such as plasma 1,25-dihydroxyvitamin D, urine calcium, and urine citrate levels, can be influenced by inherited gene mutations.^13^ PP_i_ is an inhibitor of urine calcium phosphate and calcium oxalate crystal levels.^14^^,^^15^ Hypopyrophosphaturia is associated with nephrolithiasis in *Abcc6* homozygous knockout mice.^16^^,^^17^ Biallelic deleterious mutations in *ABCC6* cause PXE, which is associated with nephrolithiasis and accelerated Randall’s plaque formation.^4^^,^^18^ Though mice with heterozygous mutations do not appear to develop calcium deposits,^19^ subclinical peripheral calcifications, including within the kidney, have been observed in humans.^5^, ^6^, ^7^ However, an association with heterozygous variants and nephrolithiasis has not previously been described. Patient 3 had a heterozygous “pathogenic” mutation (c.3413G>A (p.Arg1138Gln)) that has been observed at a frequency of < 0.01% (21/282,674 alleles) in the Broad gnomAD data set and was previously described in multiple cases of PXE in individuals with a second deleterious *ABCC6* variant.^2^^,^^19^, ^20^, ^21^ Interestingly, this variant was demonstrated to reduce ABCC6 functional transport capacity with abnormal cellular localization in the mouse liver.^20^^,^^22^ Additionally, the functional importance of this amino acid is supported by other variants, p.Arg1138Trp and p.Arg1138Pro, known to cause PXE.^2^^,^^21^^,^^23^ The point mutation reported for patient 1 (c.1685T>C (p.Met562Thr)) has been reported in the compound heterozygous state in an individual with PXE.^24^ The c.933C>A (p.Phe311Leu) mutation in patient 2 has not been implicated to cause PXE, and from a classification standpoint, the clinical impact of this variant remains inconclusive in the absence of additional supportive evidence of pathogenicity beyond the computational analysis. However, the overall wide heterogeneity of pathogenic *ABCC6* mutations^24^ that can cause PXE and the severity of his stone burden without overt risk factors except for a positive family history, strongly suggests this VUS remains relevant to the clinical presentation. The variants for patients 1 and 2 are absent from the Broad gnomAD data set suggesting these are novel variants that are not otherwise observed in healthy individuals. Although patients 1 and 3 had elevated 1,25-dihydroxyvitamin D levels, this is unlikely to be the sole etiology of their recurrent nephrolithiasis because hypercalcemia and overt hypercalciuria were absent.^25^ Patient 1 had intermittent borderline hypercalciuria but did not have any clinical signs or symptoms of an underlying granulomatous disease. Therefore, we hypothesize that heterozygous *ABCC6* variants are clinically relevant and result in hypopyrophosphaturia, thereby increasing the risk of recurrent nephrolithiasis. Although 2 of the *ABCC6* variants are classified as VUS at the time of reporting, variant classification is not static and has the potential to evolve with additional evidence over time. This was demonstrated with patient 3 with the c.3413G>A variant, which was upgraded from “likely pathogenic” to “pathogenic” after review of additional interim evidence. Of note, pseudogene interference involving exons 1-9 of *ABCC6* can impair the detection of a second variant with the Natera Renasight kidney gene panel.

Urinary PP_i_ level is not currently measured in clinical laboratories, and our hypothesis is unable to be tested at this time. A multi-institutional effort is underway to establish a clinical assay to measure urinary PP_i_ levels from patients with *ABCC6* mutations. Development of clinical assays to measure urinary PP_i_ levels may help identify people at risk of nephrolithiasis, elucidate underlying mechanisms of recurrent nephrolithiasis, and potentially identify a therapeutic target to reduce stone burden. Treatments for PXE are limited and ineffective. PP_i_ has poor oral bioavailability, but a phase 2 study looking at oral formulations with longer half-lives is in progress (NCT04868578). Additionally, INZ-701, a recombinant ENPP1 fusion protein, is in clinical development for the treatment of ectopic calcification in patients with ENPP1 or ABCC6 deficiencies (NCT05030831, NCT04686175, NCT06462547, NCT06046820, and NCT05734196). The threshold to perform genetic testing should be lowered for patients with recurrent nephrolithiasis without clear causes, given the increasing availability and affordability of these tests.^26^
